# Score for lung adenocarcinoma in China with EGFR mutation of exon 19: Combination of clinical and radiological characteristics analysis

**DOI:** 10.1097/MD.0000000000012537

**Published:** 2018-09-21

**Authors:** Zhang Shi, Xuan Zheng, Ruifeng Shi, Changen Song, Runhong Yang, Qianwen Zhang, Xinrui Wang, Jianping Lu, Yongwei Yu, Tao Jiang

**Affiliations:** a Department of Radiology; b Clinical Nutrition Department, Changhai Hospital, Second Military Medical University, Shanghai; c Department of Radiology, Yanan University Affiliated Hospital, Shanxi; d Department of Pathology, Changhai Hospital, Second Military Medical University, Shanghai, China.

**Keywords:** adenocarcinoma, epidermal growth factor receptor (EGFR), gene mutation, lung cancer, scoring model

## Abstract

**Backgroud:**

The biopsy samples might be the only tumor material available for testing the EGFR mutation status in some cases, but these samples are often composed of variable ratios of tumor to normal cells. In this study, we sought to build a scoring system to predict Epidermal growth factor receptor (EGFR) exon 19 mutation in lung adenocarcinoma by clinical and radiological features.

**Methods:**

Enrolled in this study were 601 patients with lung adenocarcinoma. Qualitative evaluation of the clinical and radiological features included 25 aspects. Statistical analysis was used to assess the association of these features between the EGFR wild type and exon 19 mutation, based on a clinical scoring system built by the statistical model and the experience of the radiologists.

**Results:**

EGRF-exon-19-mutation was associated with the female gender [odds ratios (OR), 2.573; 95% confidence intervals (CI), 1.689–3.920], tumor maximum diameter (OR, 0.357; 95% CI, 0.235–0.542), the absence of emphysema (OR, 0.202; 95% CI, 0.110–0.368), the absence of fibrosis (OR, 0.168; 95% CI, 0.083–0.339), and pleural retraction (OR, 2.170; 95% CI, 1.434–3.285). The clinical scoring model assigned 3 points to the female gender, 2 points to small tumor maximum diameter (≤34.5 mm), 2 to the absence of emphysema, 2 to the absence of fibrosis, and 1 to the presence of pleural retraction.

**Conclusions:**

The scoring system based on the statistical analysis of clinical and radiological features may be a new alternative to the prediction of EGFR mutation subtypes.

## 1 Introduction

Lung cancer is the leading cause of cancer-related death worldwide,^[1]^ and approximately 20% lung adenocarcinoma patients are epidermal growth factor receptor (EGFR) mutant. EGFR mutation is demonstrated to be higher than 60% in no-smoking persons and Asian populations.^[2]^

As is reported, patients with EGFR mutation have distinct clinical features as compared with those without the mutation.^[3]^ Some earlier studies showed that several demographic or clinical factors were associated with a high prevalence of EGFR mutations, such as the female gender, nonsmokers, and East Asian origin.^[4]^ Additionally, several articles have reported a correlation between CT features and the EGFR mutation status in nonsmall cell lung cancer (NSCLC).^[5]^ Some studies have found that higher frequencies of ground glass opacity (GGO) components^[6]^ and air bronchograms are more likely to be EGFR mutation,^[7]^ and others have found other CT features that may be associated with EGFR mutation, including air bronchograms, pleural retraction, small lesion size, and the absence of fibrosis. In addition, some researchers^[8]^ supposed that determination of the association between the CT-based radiomic features and the EGFR mutation status could provide a useful clinical predictor in patients with unresectable lung cancer or in whom biopsy was unacceptable. However, none of these variables could be adequately predicted and the findings are inconsistent with each other, although individual clinical and radiological variables are similarly associated with EGFR mutation.^[2]^

On the basis of classical studies,^[9]^ EGFR mutation includes 3 types: point mutation, multinucleotide in-frame deletion, and in-frame insertion, all of which have been documented in exon 18 through 21, highlighting that exon 19 deletion mutation (45%) is the most common mutation in lung adenocarcinoma.^[10]^ Over the past decade, the concept of targeted therapy in lung cancer has been promoted by the discovery of activating mutations in the tyrosine kinase domain of EGFR,^[11,12]^ while the EGFR tyrosine kinase inhibitors (EGFR-TKIs) have been accepted as the first targeted drugs used for the treatment of NSCLC.^[11]^ In addition, the Iressa Pan-Asia Study (IPASS) has for the first time confirmed that EGFR exon 19 and 21mutations are the strongest predictive biomarkers for progression-free survival (PFS) and tumor response to first-line gefitinib versus carboplatin/paclitaxel,^[13]^ and the researchers recommend that patients with lung cancer harboring an EGFR exon 19 mutation should be considered TKI sensitive and best managed with TKI therapy.^[14]^

However, biopsy samples might be the only tumor material available for testing the EGFR mutation status in some cases, but these samples are often composed of variable ratios of tumor to normal cells. In this study, we sought to establish a novel scoring system to predict EGFR subtype mutation in lung adenocarcinomas in a Chinese cohort of patients by using multiple clinical and radiomic features.

## 2 Materials and methods

### 2.1 Patients

A total of 1691 patients with NSCLC who underwent EGFR mutation tests between June 2011 and June 2016 in Changhai Hospital (Shanghai, China) were initially enrolled in this study, from whom 742 patients were selected according to the following inclusion criteria^[15]^: patients with pathologically confirmed diagnosis of lung adenocarcinoma and underwent EGFR mutation test in our hospital; and patients with preoperative thin-section CT images accessible in our picture archiving and communication system (PACS). Of the 742 patients, 141 patients were finally excluded from the study according to the following exclusion criteria: CT scan performed at another institution or not including the chest at our institution; patients who did not undergo surgery; and patients with the EGFR mutation subtype not in exon 19. Finally, 601 ethnically Chinese patients were reserved for analysis. The study protocol was approved by the Ethics Committee of Changhai Hospital and the Institutional Review Board of the Second Military Medical University (Shanghai, China; the clinical trial registration number: ChiCTR-DOD-15005777).

### 2.2 Molecular profiling

Tumor specimens for EGFR mutation analysis were obtained from surgical resection. EGFR mutation analyses in the tyrosine kinase domain (exons 19) frequently seen in lung adenocarcinoma were performed.^[8]^ Tumors were diagnosed as adenocarcinoma and classified according to the 2015 WHO Classification.^[16]^ EGFR-wild type and EGFR-mutated subtypes were determined by an amplification refractory mutation system real-time technology using Human EGFR Gene Mutations Fluorescence Polymerase Chain Reaction (PCR) Diagnostic Kit (Amoy Diagnostics Co., Ltd, Xiamen, China).^[15]^

### 2.3 CT

CT examinations were randomly performed on 2 16-slice Philips CT systems (Philips, Brilliance-16 and MX-16, the Netherlands), a 64-slice Siemens system (Siemens, Sensation Cardiac 64, Germany) or a 320-slice CT system (Toshiba, Aquilion ONE, Japan). All examinations were extended in a craniocaudal direction, with or without contrast medium. All images were archived in a digital format.

All qualitative image analyses were performed by 3 senior radiologists with more than 20-year experience in the diagnostics of thoracic imaging, who were blind to the EGFR genomic classifications. Discrepancies in interpreting the CT features between them were resolved by discussion until consensus was reached. According to the date of clinical features and CT examinations, each patient was extracted from the medical records. For each patient, 23 factors of the radiological features from the CT examinations were recorded on an Excel file (Microsoft Office Excel 2013) as shown in Table 1, which included maximum diameter (mm) of the lesion; spot of the lesion; shape; margins; the presence or absence of a ground-glass opacity (GGO); lesion density; lesion with or without vacuole sign; the presence or absence of cavitation; the presence or absence of air bronchograms; thickening of the adjacent pleura; the presence or absence of necrosis in the tumor; the presence or absence of satellite nodules in the primary tumor lobe; the presence or absence of nodules in nontumor lobes; the presence or absence of pleural retraction; location of the lesion, including central and peripheral; the presence or absence of intra-nodular calcifications; the presence or absence of emphysema; the presence or absence of fibrosis; the presence or absence of pleural contact; the presence or absence of metastases; the presence or absence of pulmonary hilar lymph node enlargement; the presence or absence of mediastinal lymph node enlargement; the degree of contrast enhancement (indicated as no enhancement 15–30 HU, 30–50 HU, 50–70 HU, >70 HU).

**Table 1:**
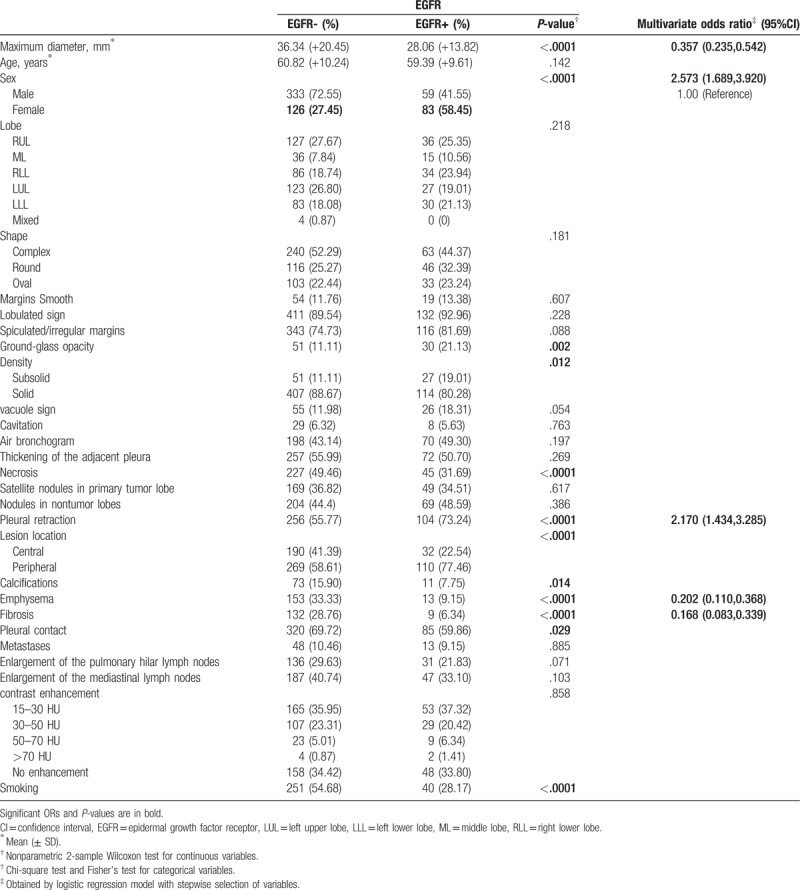
Univariate and multivariate analyses of the EGFR wild type and EGFR mutation in exon 19.

### 2.4 Statistical analysis

All statistical analyses were performed using SPSS 21.0. As the age and maximum diameter were suggested as a normal distribution by SPSS software, *t*-test was performed to analyze these variables. Nonparametric 2-sample Wilcoxon test was used for continuous variables (such as lesion density) and order variables (the degree of contrast enhancement), and chi-square test and Fisher's test for categorical variables. Subsequently, multivariate analysis was performed to establish a logistic regression model with stepwise selection of variables. As per stepwise selection, effects were entered into and removed from the model. Thus, one or more backward elimination steps could follow each forward selection step. At each forward selection step, if it was significant at the *P = *.05 level, the corresponding effect was added to the model. Meanwhile, results of the Wald test for individual parameters were examined at each backward elimination step. The least significant effect not meeting the *P = *.05 level was removed. The stepwise selection process terminated when no further effect could be added to the model or when the current model was identical to a previously visited model. The linear combination of the regression coefficients of the exponential component of the final multiple logistic regression model was used to compute a prognostic score, from this point onward referred to as the statistically determined prognostic score.^[17]^ ROC curves were drawn for EGFR exon 19 mutation according to their significant characteristic, and then the corresponding area under the curve (AUC) was calculated. *P*-values < .05 were considered statistically significant. The clinical scoring model was proposed on the base of the statistical model and the experience of the radiologists. And the ROC curves for the statistically determined prognostic scoring model and the clinically specified prognostic scoring model were plotted together on the same picture. The respective AUC values were also calculated.

## 3 Results

**Figure 1:**
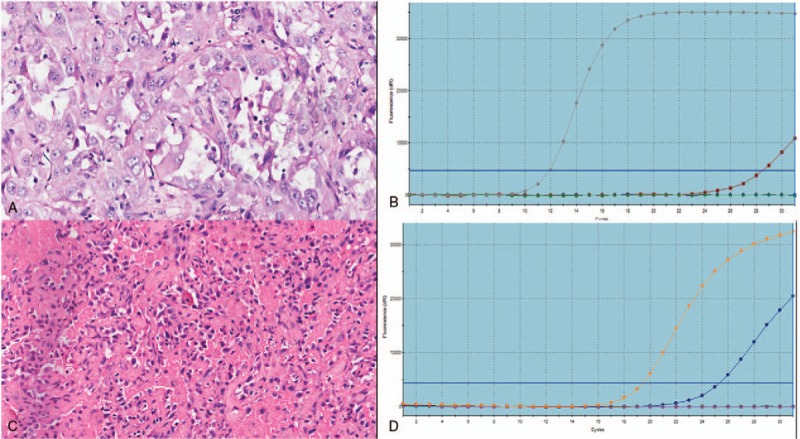
The pathological photo (A, C) and PCR picture (B, D) of the EGFR wild type (A, B) and the exon 19 delection (C, D), respectively.

**Figure 2:**
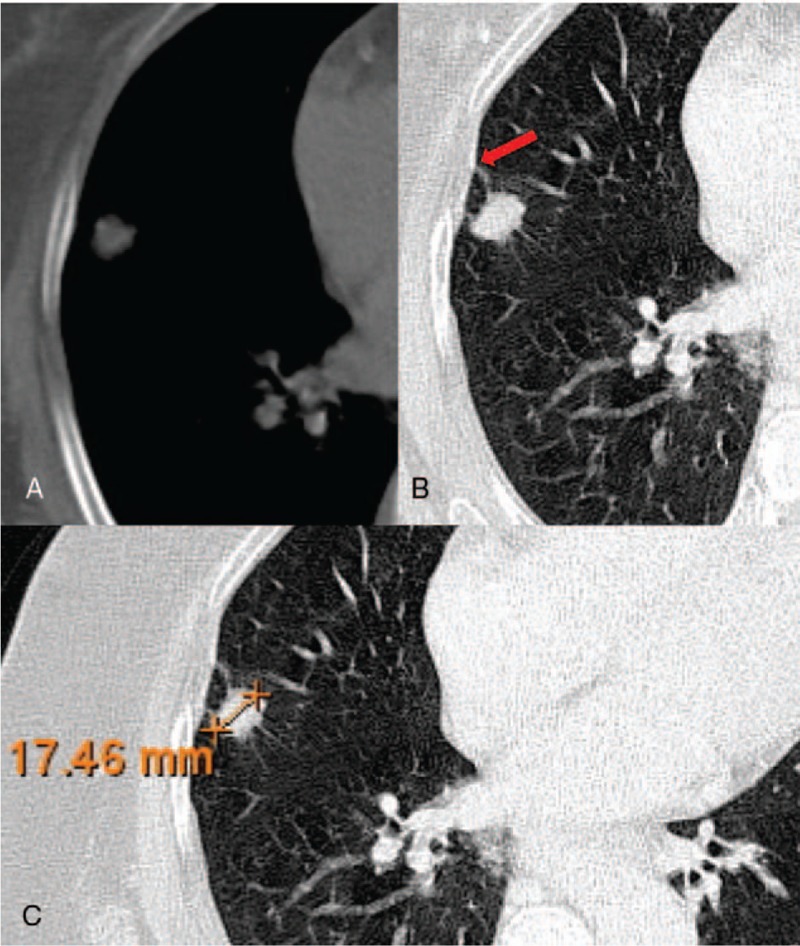
A 69-year-old woman with EGFR-mutated in exon 19 of lung cancer in the right upper lobe, and CT images show a small maximum-diameter mass about 17 mm (C) with obvious pleural retraction (B, red arrow), the absence of emphysema and fibrosis (A). EGFR = epidermal growth factor receptor.

**Figure 3:**
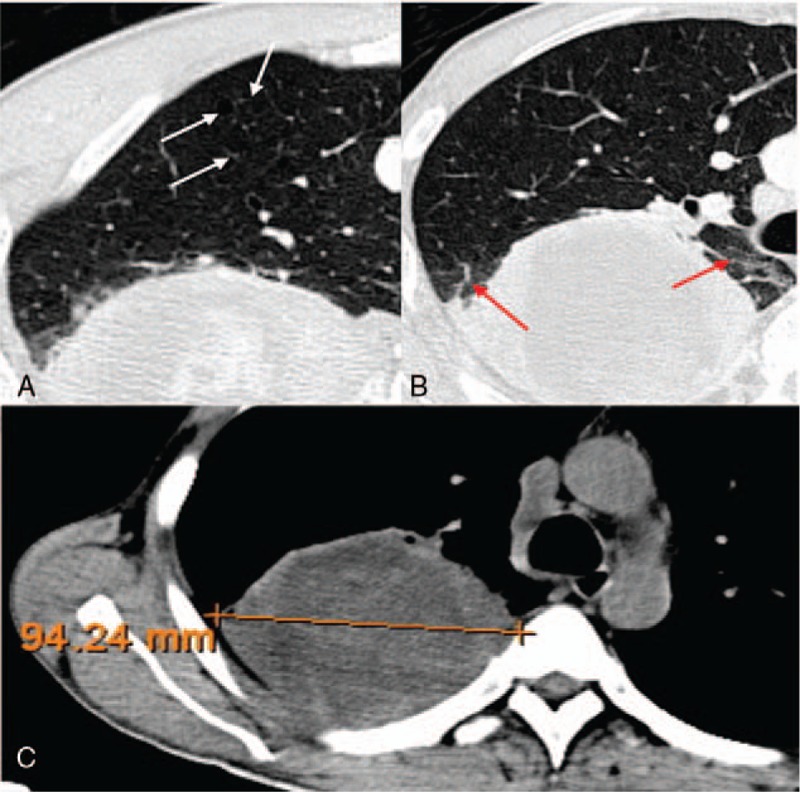
A 55-year-old man with a smoking history of 35 years had right upper lobe lung adenocarcinoma in EGFR wild type, in whom axial CT images show a solid mass with some emphysema (A, white arrow), a litte fibrosis (B, red arrow) and no pleural retraction or Spicule sign, whose maximum diameters were about 94 mm (C).

According to the inclusion and exclusion criteria, of the 601 included patients, 142 patients (mean age 59.39 ± 9.61 years; M:F = 59:83) exhibited EGRF exon 19 mutation (Fig. 1) and 459 patients (mean age 60.82 ± 10.24 years; M:F = 333:126) exhibited EGFR wild type (Fig. 2). CT and clinical characteristics of EGFR mutations are summarized in Table 1. As shown in Table 1, univariate analysis showed that 12 characteristics could be used to help identify EGFR exon 19 mutation. Multiple logistic regression analysis showed that sex [odds ratio (OR), 2.573; 95% CI, 1.689–3.920], emphysema (OR, 0.202; 95% CI, 0.110–0.368), maximum diameter (OR, 0.357; 95% CI, 0.235–0.542), the fibrosis (OR, 0.168; 95% CI, 0.083–0.339) and pleural retraction (OR, 2.170; 95% CI, 1.434–3.285) were important predictors of EGFR exon 19 mutation, where the AUC of ROC was 0.655, 0.621, 0.618, 0.612, and 0.587, respectively (Fig. 3). According to the result of statistical analysis, the cut-off value of the maximum diameter was 34.5 mm, indicating that tumors with a maximum diameter smaller than 34.5 mm were more likely to harbor EGFR exon 19 mutation.

**Figure 4:**
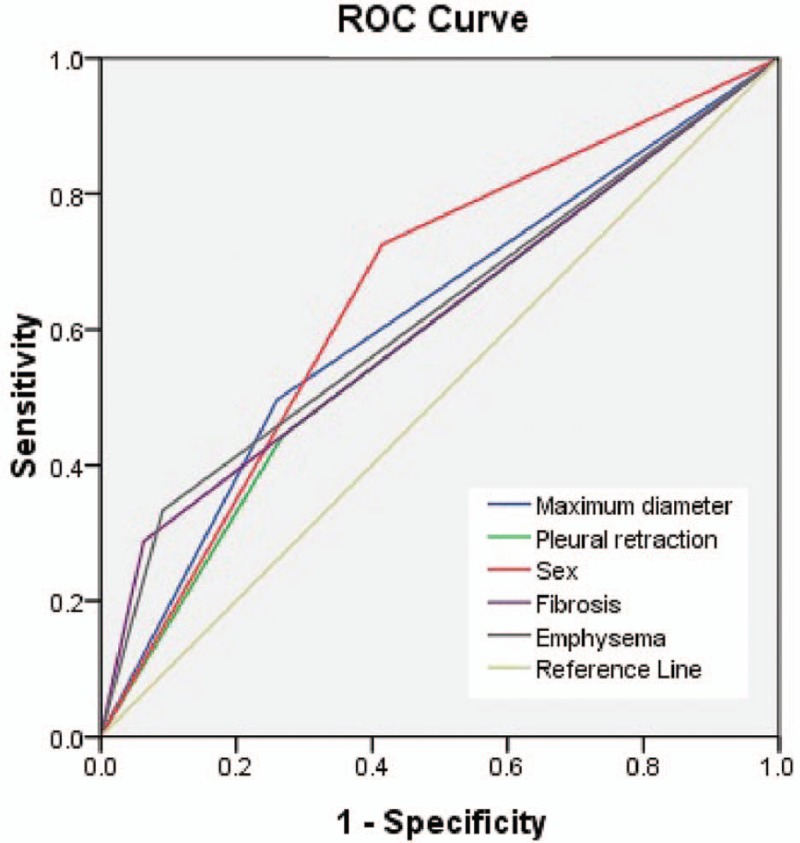
ROC curves for EGFR mutation in exon 19 by the univariate analysis. The AUCs of ROCs were 0.655 (sex), 0.621 (emphysema), 0.618 (maximum diameter), 0.612 (the fibrosis), and 0.587 (leural retraction). AUC = area under the curve, EGFR = epidermal growth factor receptor, ROC curve= receiver operating characteristic curve

According to the results of multivariate logistic regression analysis, a statistically determined prognostic model of EGFR exon 19 mutation could be built by the following equation: 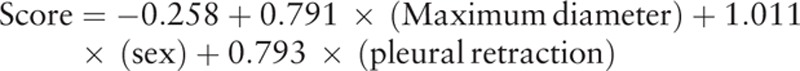 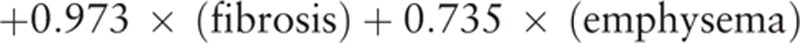


These results could be used for building a scoring system that is more convenient and more practical for clinical prediction, in which 3 points was assigned to the female gender, 2 points to tumors with a small maximum diameter ≤34.5 mm, 2 points to the absence of emphysema, 2 points to the absence of fibrosis, and 1 point to tumors with pleural retraction. The scoring model is specified by the following equation: 
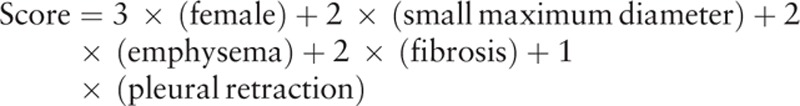


The clinical scoring model for each of the prognostic factors is shown in Table 2. The ROC curves of the statistically determined scoring model and the clinical scoring model are presented in Figure 4, whose AUC values were 0.753 and 0.755, respectively. When a total clinical score was 3.5 (the cut-off value), the sensitivity of the clinical scoring system was 63.0% and the specificity was 76.1%. When the total score was up to 7.5, the specificity was higher to 98.6% ().

**Table 2:**
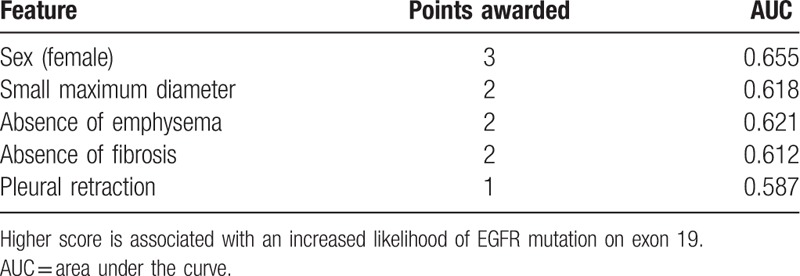
Clinical scoring model.

**Figure 5:**
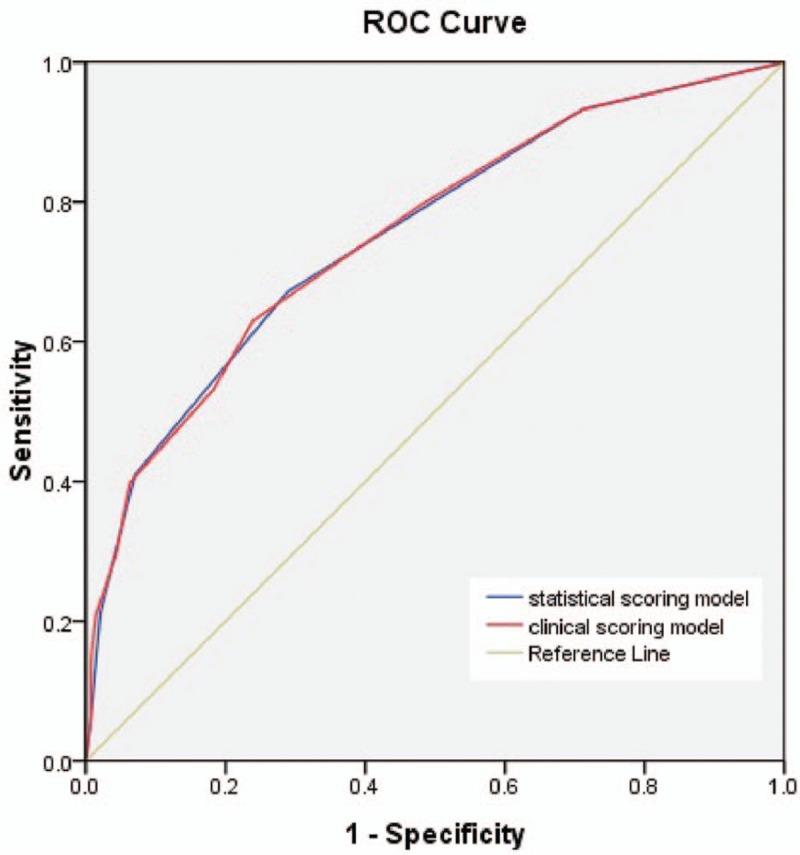
ROC curves for the statistical scoring model and the clinical scoring model. The AUCs of ROC curves about the statistically determined scoring model and the clinical scoring model were 0.753 and 0.755, respectively. AUC = area under the curve, ROC curve= receiver operating characteristic curve.

Based on the above results, a clinical scoring model was established for predicting the mutation subtypes and distinguishing between the EGFR wild type and EGFR exon 19 mutation. The model clearly indicated that female patients with lung adenocarcinoma whose lesions were relatively smaller with less fibrosis and emphysema and more pleural retraction would have a higher correlativity to exon 19 mutation.

## 4 Discussion

The results of the present study demonstrated that the scoring system based on the clinical and radiological features could help distinguish between EGFR exon 19 mutation and EGFR wild type. Compared with EGFR wild type, EGFR exon 19 mutation has some distinct clinicoradiologic characteristics, including the female gender, pleural retraction, small lesion diameter, the absence of emphysema and fibrosis. In addition, this scoring model performed better than individual demographic or radiological features in predicting EGFR exon 19 mutation. The scoring model is less complex so that it could be performed after a CT examination without additional other tests.

Compared with previous demographic analyses indicating that the female gender, adenocarcinoma histology, the nonsmoking status and Asian ethnicity are the most significant factors associated with EGFR mutations and response to EGFR-TKIs,^[18]^ we found that the female gender was more closely associated with EGFR exon 19 mutation. Our finding is also consistent with Sabri et al,^[17]^ who reported that the female gender was an independent predictor of EGFR mutation [OR = 2.944

(1.055–8.221)] and more likely to have EGFR mutation-positive tumors (71% of the EGFR-positive tumours were found in women vs 46%of the EGFR-negative tumours). What is more, the article by Cao et al^[19]^ showed that female and smoking were significantly associated with EGFR exon 19 mutation, which was the similar as our study.

In addition, we found that lung adenocarcinoma with EGFR exon 19 mutation had more specific CT features (small lesion diameter, pleural retraction, and the absence of emphysema and fibrosis) as compared with EGFR wild type. The relatively small maximum diameter in patients with EGFR exon 19 mutation as obverted in this study is supported by the prior research by Hsu et al^[20]^ who reported that adenocarcinomas with EGFR exon 19 mutation were significantly associated with small tumors. What is more, the other 3 radiological features (pleural retraction and the absence of emphysema and fibrosis) observed in this study are also consistent with other recent studies,^[15,17]^ which reported that pleural retraction as a frequent sign of visceral pleural invasion is one of the most important prognostic factors in patients undergoing complete resection for NSCLC.^[21]^

Although there have been many studies about the clinical and radiological features in EGFR mutations, they only described the clinicoradiological association with the whole types of EGFR mutation without addressing the characteristics of EGFR mutation subtypes. However, none of them reported associations between the radiological features and EGFR mutation subtypes, while all the above studies discussed the correlation of the radiological features with the diagnosis of EGFR-mutated lung cancer.

Classically, EGFR belongs to the ERBB family of cell-surface tyrosine kinase receptors.^[22]^ EGFR is mutated in about 16% tumor specimens from patients with NSCLC.^[23]^ There are several described mutations in the EGFR gene, in which the 2 most common are short in-frame deletions around the LREA motif of exon 19 (45%–50%) and a point mutation (CTG to CGG) in exon 21, resulting in substitution of leucine by arginine at codon 858, L858R (45%–50%).^[24]^ Differences between the subtypes of EGFR-mutated genes result in the discrepancy of the coding protein and the diversity of targeted treatment. Mutations in exon 19 and 21 are responsible for 90% EGFR mutations in lung adenocarcinoma and sensitive to the targeted drugs.^[25]^ Recently, some studies found that there was little difference in the treatment and prognosis between exon 19 deletion and exon 21 mutation. A meta-analysis by Zhang et al^[26]^ indicated that exon 19 deletion might be associated with longer PFS compared with EGFR exon 21 mutation after administration of first-line EGFR-TKIs for NSCLC patients. Similarly, 2 reports by Liu et al^[27]^ and Sheng et al^[28]^ suggested that NSCLC patients with EGFR exon 19 deletion had a longer PFS and OS, and a higher response rate after EGFR-TKI therapy compared with those with exon 21 L858R mutation. As the latest study described, early radiological response could identify a subgroup of patients with EGFR mutation, and patients with exon 21 L858R had a poor prognosis in spite of the treatment with TKI.^[29]^ Therefore, it is strongly recommended to identify the subtype of EGFR mutation in the clinical treatment of lung cancer.

In this study, we further probed the association between the EGFR wild type and EGFR exon 19 mutation, and established a scoring model for primary prediction to help first-step diagnosis of lung cancer. Analysis of the radiological features showed that EGFR exon 19 mutation was associated with a small maximum diameter, pleural retraction and the absence of emphysema and fibrosis, which are similar to the report of Liu et al.^[15]^ The present study also established an equally important clinical scoring system to predict EGFR exon 19 mutation. Theoretically, this clinical scoring system seems more scientific to predict the EGFR mutation as compared with scoring model that was based on the clinical and radiological features only. Actually, although the ROC curves of the 2 scoring models are almost the same, the 10-point clinical scoring model seems more reasonable and customary to be used for clinicians as compared with the 5-point model based on statistical analysis according AUC. Fortunately, the specificity of the total score up to 7 in our scoring system is higher than the other scoring model reported in another recent study.^[17]^ Of course, this conclusion needs to be confirmed by larger sample studies. Therefore, 142 EGFR mutations in exon 19 were chosen from approximately 601 cases of lung adenocarcinoma, and the finding that the scoring system about the distinction in EGFR mutation subtypes could be helpful and useful for selection of suitable clinical treatments. Compared with clinical examinations such as direct sequencing of PCR-amplified genomic DNA, high-resolution melting analysis, fragment analysis, and the amplification refractory mutation system,^[30]^ which are generally expensive and sometimes do not have a high rate of tumor cell detection, the scoring model described in this can not only discriminate EGFR-mutated subtypes (exon 19 deletion) but also are noninvasive and less expensive, especially for advanced NSCLC patients who cannot receive biopsy.^[31]^

To the best our knowledge, this is the first study describing a comprehensive scoring system to predict EGFR exon 19 mutation. Compared with the recent similar report,^[17]^ our study paid more attention to the distinction between EGFR wild types and EGFR-mutated subtypes, which should be helpful and useful for clinical treatment. Nonetheless, there are some limitations in this study. First, this study is limited to Chinese populations only. Second, patients with other EGFR mutation subtypes were not included in our study, and larger patient cohort studies are required to confirm our observation. Finally, the scoring system obtained from the retrospective analysis in this study needs to be further confirmed by prospective studies including nonsurgical candidates and different ethnic populations to determine whether this model can be used for treatment decision-making without molecular profiling.^[17]^

## 5 Conclusion

This clinical and radiological analysis of EGFR revealed certain associations between the EGFR wild status and EGFR exon 19 mutation (the female gender, pleural retraction, a small lesion diameter, the absence of emphysema and the absence of fibrosis), and a scoring system based on this multivariable analysis may suggest a new proposal to predict the EGFR mutation status. The scoring system including the CT imaging features of lung adenocarcinomas in combination with clinical variables may prove to be useful in prognosticating EGFR mutation subtypes.

## Acknowledgments

We would like to thank the Ethics Committee of Changhai Hospital and the Institutional Review Board of the Second Military Medical University for the approval of Ethical compliance. We also thank Prof. Jiang (Department of Radiology, Changhai Hospital) for the guarantor of the entire study, and thank Prof. Lu (Department of Statistics, Second Military Medical University) for the statistical advice. Lastly, we thank the National Natural Science Foundation of China (No. 81402680, No. 81371551), Shanghai Rising-Star Program (12QA1404700), Special program of military medicine of second military medical university (2011JS18), Changhai Hospital 1255 Scientific Innovation Funds (CH125541000) for the financial supports.

## Author contributions

**Data curation:** Zhang Shi, Ruifeng Shi, Runhong Yang, Changen Song, Qianwen Zhang, Xinrui Wang.

**Formal analysis:** Ruifeng Shi, Changen Song, Qianwen Zhang, Xinrui Wang, Yongwei Yu, Jianping Lu, Tao Jiang.

**Funding acquisition:** Jianping Lu, Tao Jiang.

**Methodology:** Xuan Zheng.

**Resources:** Xuan Zheng.

**Software:** Yongwei Yu.

**Validation:** Zhang Shi, Yongwei Yu, Jianping Lu, Tao Jiang.

**Visualization:** Tao Jiang.

**Writing – original draft:** Zhang Shi, Xuan Zheng.

**Writing – review & editing:** Zhang Shi.

Zhang Shi orcid: 0000-0001-7739-7497
